# Optic Foraminotomy for Clipping of Superior Carotid-Ophthalmic Aneurysms

**DOI:** 10.3389/fsurg.2021.681115

**Published:** 2021-12-09

**Authors:** Matias Baldoncini, Sabino Luzzi, Alice Giotta Lucifero, Ana Flores-Justa, Pablo González-López, Alvaro Campero, Juan F. Villalonga, Michael T. Lawton

**Affiliations:** ^1^Department of Neurological Surgery, San Fernando Hospital, Buenos Aires, Argentina; ^2^Laboratory of Microsurgical Neuroanatomy, Second Chair of Gross Anatomy, School of Medicine, University of Buenos Aires, Buenos Aires, Argentina; ^3^Neurosurgery Unit, Department of Clinical-Surgical, Diagnostic and Pediatric Sciences, University of Pavia, Pavia, Italy; ^4^Neurosurgery Unit, Department of Surgical Sciences, Fondazione IRCCS Policlinico San Matteo, Pavia, Italy; ^5^Department of Neurosurgery, University General Hospital of Alicante, Alicante, Spain; ^6^Department of Neurological Surgery, Padilla Hospital, Tucumán, Argentina; ^7^Department of Neurosurgery, Barrow Neurological Institute, Phoenix, AZ, United States

**Keywords:** anterior clinoidectomy, microsurgical clipping, optic foraminotomy, optic nerve decompression, skull base, superior carotid-ophthalmic aneurysms

## Abstract

**Background:** Carotid-ophthalmic aneurysms usually cause visual problems. Its surgical treatment is challenging because of its anatomically close relations to the optic nerve, carotid artery, ophthalmic artery, anterior clinoid process, and cavernous sinus, which hinder direct access. Despite recent technical advancements enabling risk reduction of this complication, postoperative deterioration of visual function remains a significant problem. Therefore, the goal of preserving and/or improving the visual outcome persists as a paramount concern.

**Objective:** We propose optic foraminotomy as an alternative microsurgical technique for dorsal carotid-ophthalmic aneurysms clipping. As a secondary objective, the step by step of that technique and its benefits are compared to the current approach of anterior clinoidectomy.

**Methods:** We present as an example two patients with superior carotid-ophthalmic aneurysms in which the standard pterional craniotomy, transsylvian approach, and optic foraminotomy were performed. Surgical techniques are presented and discussed in detail with the use of skull base dissections, microsurgical images, and original drawings.

**Results:** Extensive opening of the optic canal and optic nerve sheath was successfully achieved in all patients allowing a working angle with the carotid artery for correct visualization of the aneurysm and further clipping. Significant visual acuity improvement occurred in both patients because of decompression of the optic nerve.

**Conclusion:** Optic foraminotomy is an easy and recommended technique for exposing and treating superior carotid-ophthalmic aneurysms and allowing optic nerve decompression during the first stages of the procedure. It shows several advantages over the current anterior clinoidectomy technique regarding surgical exposure and facilitating visual improvement.

## Introduction

Carotid-ophthalmic segment aneurysms arise from the internal carotid artery (ICA) between the distal dural ring and the origin of the posterior communicating artery at any point of its diameter (1). Aneurysms occurring in this segment are relatively rare and account for 0.5–11% of all intracranial aneurysms (2–5). A higher incidence in females and left-sided predominance of carotid-ophthalmic artery aneurysms have been reported (2, 3, 6–11). These are frequently large or giant, and there is a high association with multiple aneurysms (21–64%) (7, 8, 10, 12–15).

Carotid-ophthalmic aneurysms are globally classified as paraclinoid aneurysms, as in the Bouthillier classification of the ICA segments (16). Paraclinoid aneurysms have been widely classified by Krisht et al. in superior (true ophthalmic artery), inferior (or ventral aneurysms), lateral (subclinoid aneurysms), and medial (superior hypophyseal aneurysms and carotid cave aneurysms) (17–20).

**Figure 1:**
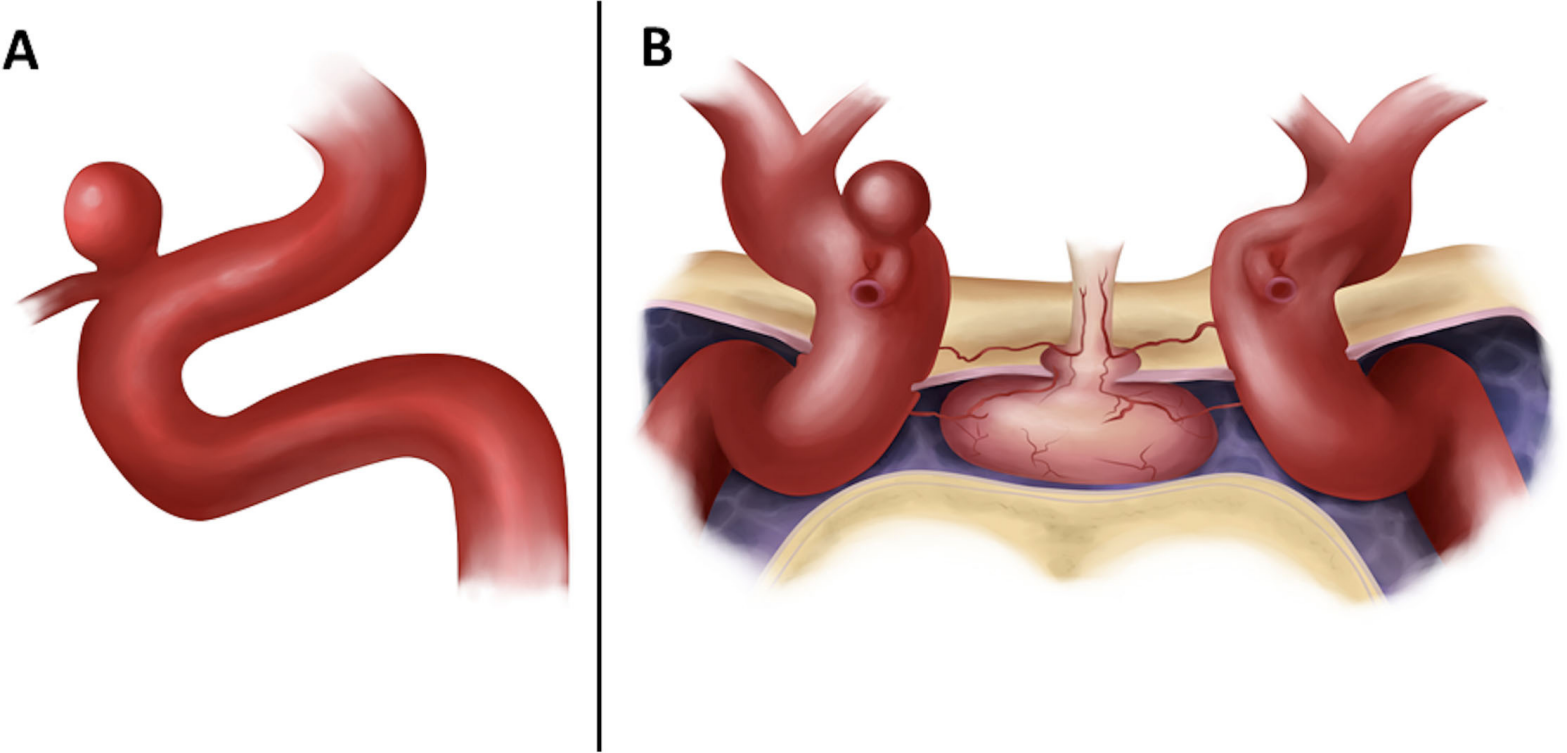
**(A)** Original drawing showing a lateral view of the cavernous, clinoid, and ophthalmic segments of the carotid artery with an aneurysm arising on the superior surface at the origin of the ophthalmic artery. **(B)** Anterior view of the sellar and parasellar region in a coronal section. We can see the intracavernous segment of both ICA and the supraclinoid segment showing in the right ICA a superior carotid-ophthalmic artery aneurysm.

Carotid-ophthalmic aneurysms usually point in the direction of the main hemodynamic force, immediately proximal to the aneurysm site, and in the direction, the blood would have flowed without considering the curve at the aneurysm site (21) (Figure 1A). The perforating branches arising from the ophthalmic segment are on the medial side of this aneurysm (21). These aneurysms often cause visual deficits due to the optic nerve compression (22). Two factors are involved in the visual symptoms' development: the direction of the aneurysm, and its overall size (2).

Direct repair of paraclinoid aneurysms is challenging because of its anatomically close relations to the optic nerve, carotid artery, ophthalmic artery, anterior clinoid process, and cavernous sinus, which hinder direct access (Figure 1B). Despite recent technical advancements enabling a remarkable risk reduction of this complication, postoperative deterioration of visual function remains a significant problem. Therefore, protection of the optic nerve must remain a paramount concern when treating paraclinoid aneurysms (23).

We report two patients with superior carotid-ophthalmic aneurysms, and we describe our results and experience with pterional craniotomy, transylvian dissection, and optic foraminotomy as an alternative microsurgical approach for clipping superior carotid-ophthalmic aneurysms and allowing optic nerve decompression. As a secondary objective, we will describe the step by step of technique with the use of skull base dissections, microsurgical images, and original drawings.

## Materials and Methods

We present two surgical interventions of superior carotid-ophthalmic aneurysms. Photographs attached in the present article were taken with a Nikon D7200 camera with a Micro Nikon 40 mm F2.8 (Nikon Corporation, Minato, Tokyo, Japan) objective and annular flash. Microsurgical dissections were performed under a ZEISS^®^ microscope (Carl Zeiss, Oberkochen, Germany) and Blackmagic^®^ (Black Magic Cinema Cameras, Port Melbourne, Victoria, Australia).

### Patients

**Figure 2:**
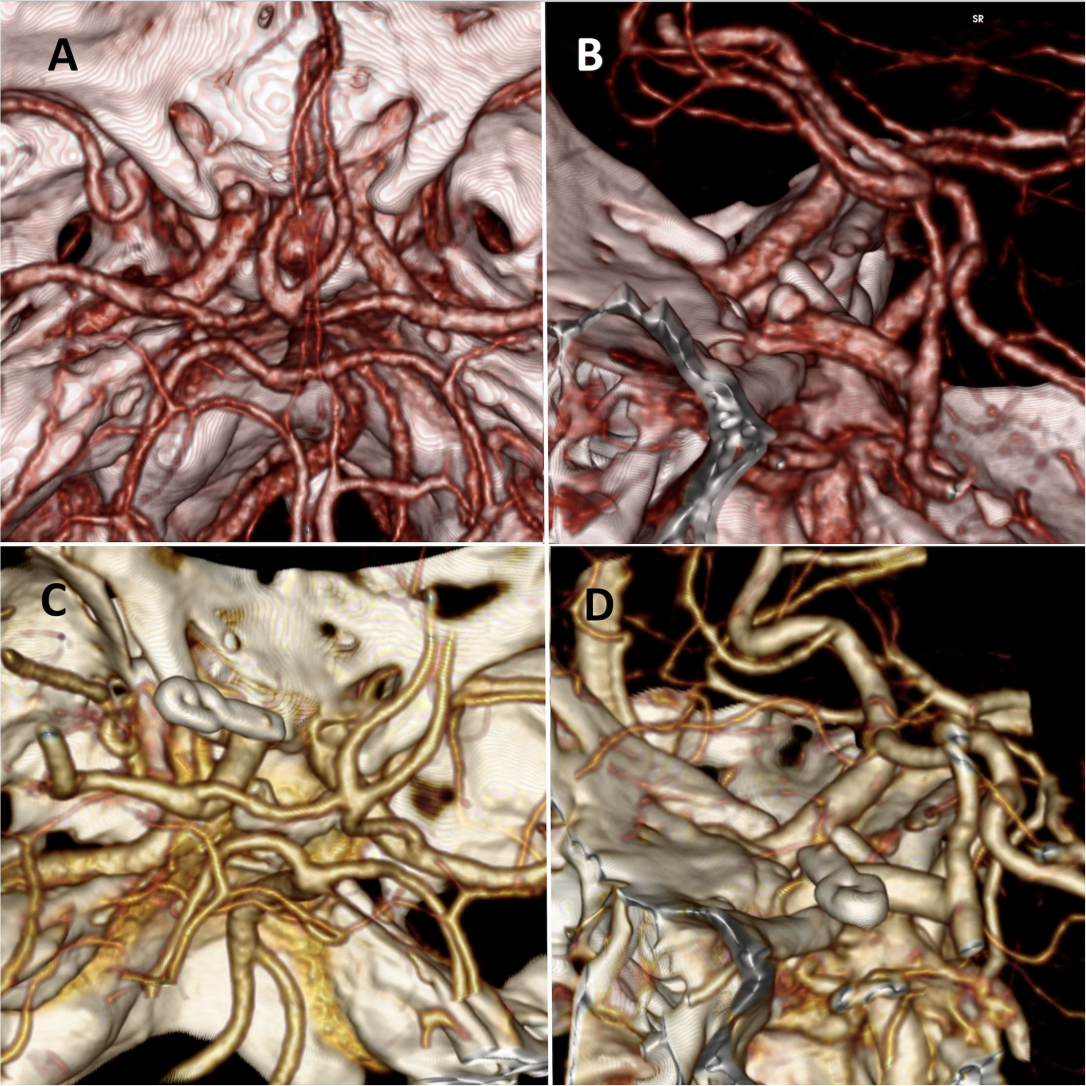
**(A,B)** Preoperative CT angiography reconstruction of case 1 where we can observe a left superior carotid-ophthalmic aneurysm. **(C,D)** Postoperative CT angiography reconstruction with and excluded aneurysm after clipping.

Patient 1: A 64-year-old man with a left inferior visual field deficit and headache. The CT angiography showed a left superior carotid-ophthalmic aneurysm (4 × 5 mm) (Figures 2A,B).

**Figure 3:**
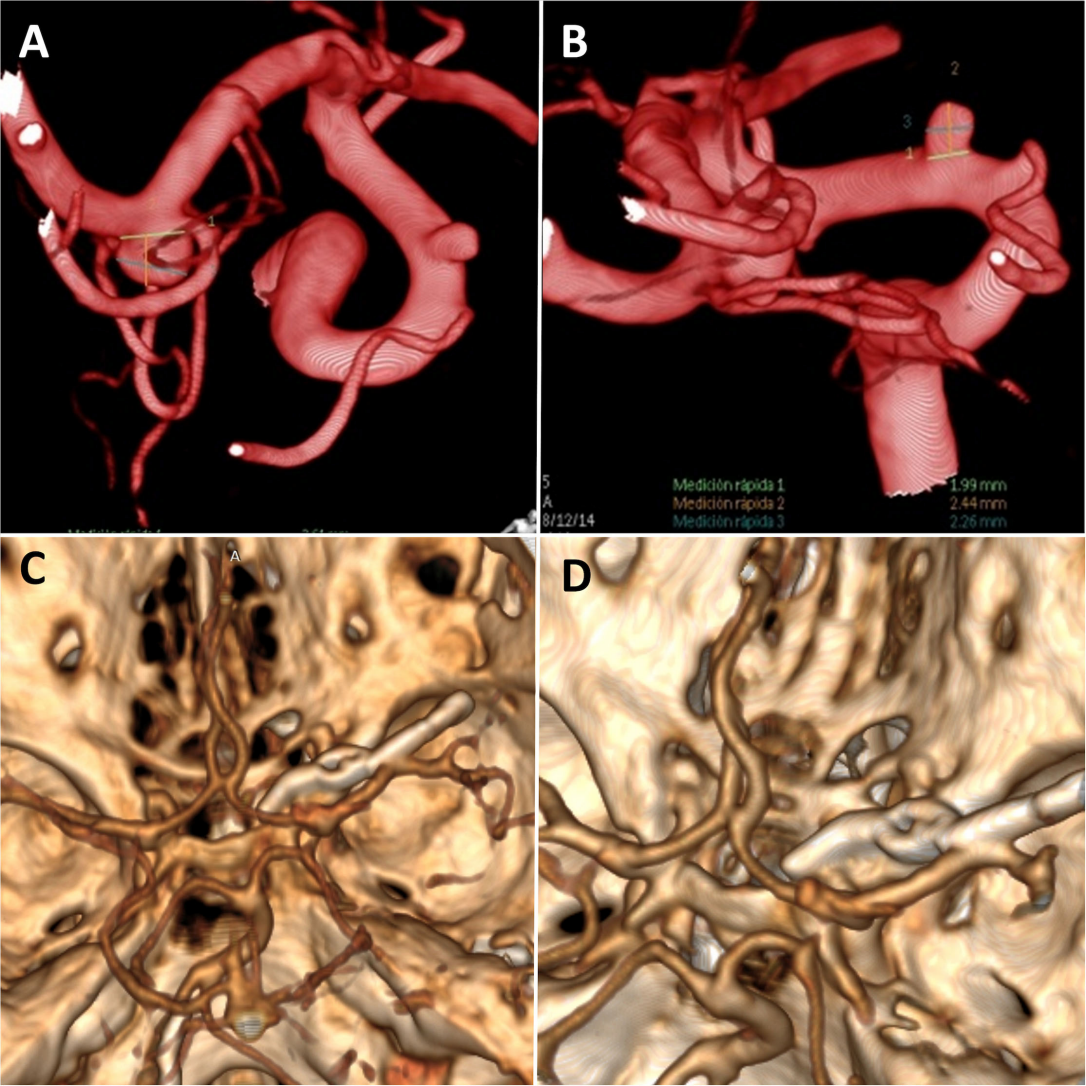
**(A,B)** Preoperative digital 3D angiographic reconstruction of case 2 where we can observe two right cerebral aneurysms; a medial cerebral artery and a superior carotid-ophthalmic aneurysm. **(C,D)** Postoperative CT angiography showing the foraminotomy and aneurysms clipped.

Patient 2: A 43-year-old woman with subarachnoid hemorrhage, classified as Fisher I, Hunt, and Hess II. In the digital 3D angiographic reconstruction multiple aneurysms are observed: a right M1 aneurysm and a right superior carotid-ophthalmic aneurysm (Figures 3A,B).

### Surgical Technique

**Figure 4:**
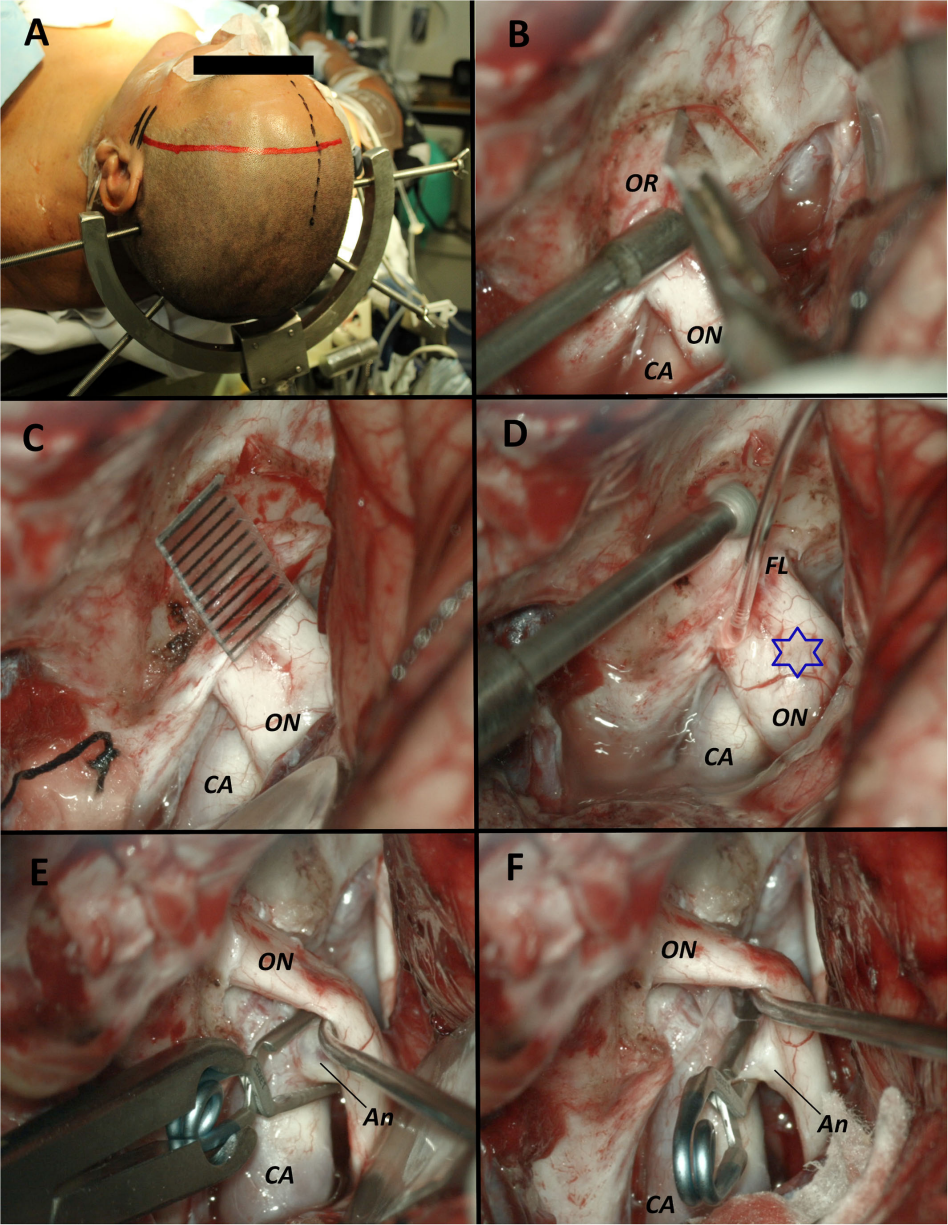
Surgical images from Case 1. **(A)** Patient position in supine with the head fixed in a 4-pin Sugita head holder and a left curvilinear frontotemporal incision is marked behind the hair line. **(B)** Exposure of the optic nerve and the carotid artery where we can observe the optic canal roof. A circumferential incision of the dura above the optic canal was made with a blade N°11. **(C)** Dissection and removal of the dura is performed with the help of a Penfield microdissector. A caliper marks a length of 10 mm from the free edge of the falciform ligament where we performed the incision of the dura around the optic canal. **(D)** Removal of the bony roof with a 3 mm diamond high speed drill under constant irrigation to avoid thermal damage to the optic nerve. The blue star marks the protrusion of the optic nerve due to the aneurysm. **(E)** Surgical view after the optic foraminotomy where the roof and both the medial and lateral walls of the optic canal were removed. The optic nerve became gently retractable with a number 7 Penfield dissector away from the carotid artery where the aneurysmal neck is easily exposed for its clipping. **(F)** Final image of the carotid-ophthalmic clipped aneurysm. Decompression of the optic nerve is demonstrated. OR, Optic roof; ON, Optic nerve; CA, Carotid artery; FL, Falciform ligament; Star, Protrusion of the optic nerve due to the aneurysm; An, Aneurysm.

**Figure 5:**
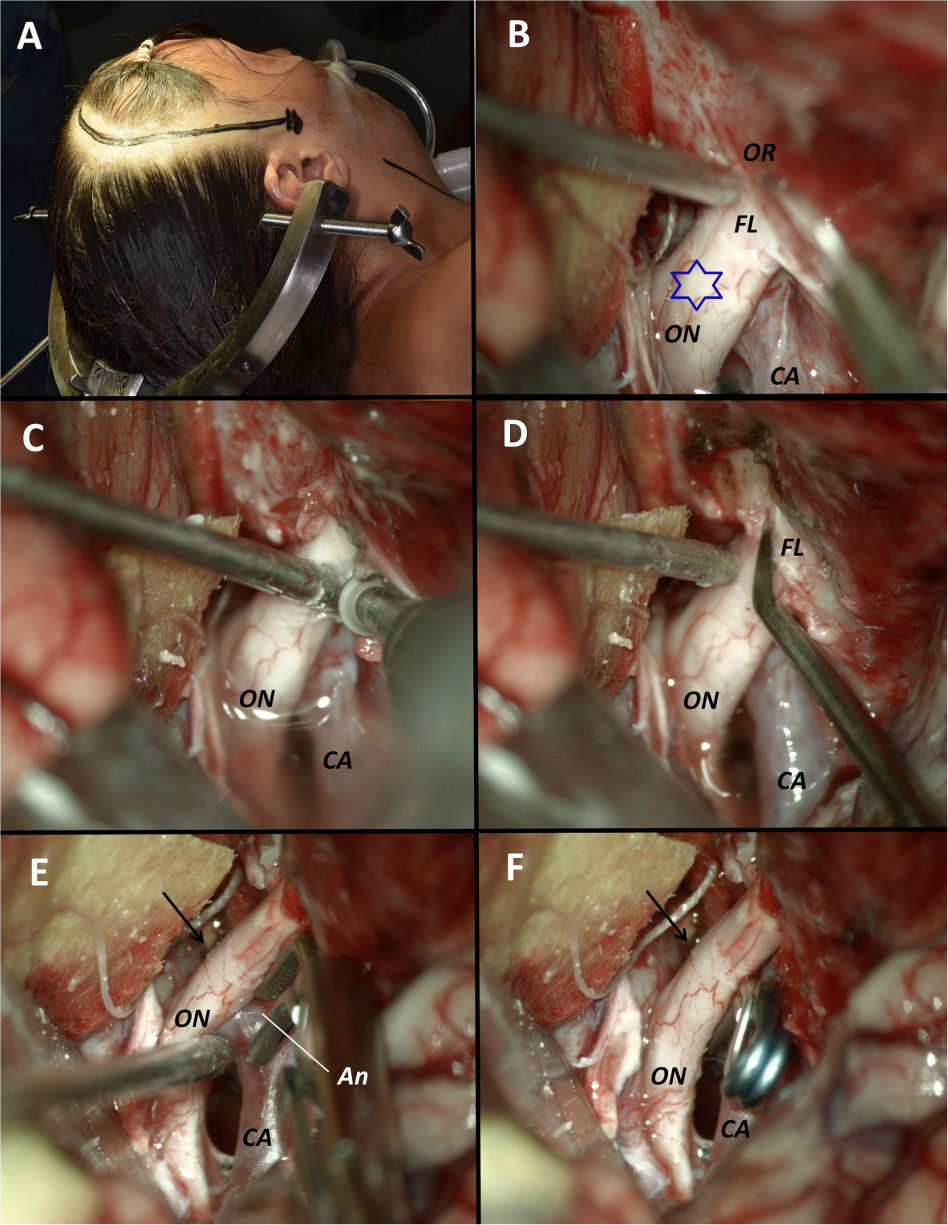
Surgical images from Case 2. **(A)** Patient position in supine with the head fixed in a 4-pin Sugita head holder and a right curvilinear frontotemporal incision is marked behind the hair line. **(B)** Exposure of the optic nerve and the carotid artery where we can observe the optic canal roof and the falciform ligament. A circumferential incision of the dura above the optic canal was made with a blade N°11. The blue star marks the protrusion of the optic nerve due to the aneurysm. **(C)** Dissection and removal of the dura is performed. Drilling of the roof, lateral and medial wall of the optic canal were performed. **(D)** Incision of the falciform ligament with an angular blade N° 11 was performed. **(E)** The optic nerve became gently retractable away from the carotid artery where the aneurysmal neck is easily exposed for its clipping. The arrow marks the level where the falciform ligament was. **(F)** Final image of the carotid-ophthalmic clipped aneurysm. Decompression of the optic nerve is demonstrated. OR, Optic roof; ON, Optic nerve; CA, Carotid artery; FL, Falciform ligament; Star, Protrusion of the optic nerve due to the aneurysm; Arrow, location where the falciform ligament was prior to the excision; An, Aneurysm.

Patients are positioned supine, with the head fixed in a 4-pin head holder with a 15/30° rotation to the contralateral side. A curvilinear-shaped frontotemporal incision behind the hairline and an interfascial dissection (24, 25) of the temporal muscle is performed. A pterional craniotomy and flattening of the sphenoid ridge with the usual drilling procedure are carried out. An arciform durotomy with the opening of the Sylvian fissure to the carotid cistern was done under the microscope. The dura of the anterior cranial base, optic nerve, carotid artery, and its bifurcation is exposed. A circumferential dural incision is made above the optic canal. The length used is around 10 mm in front of the free edge of the falciform ligament. Dissection and removal of the dura to avoid rolling in the drill and lesion to the optic nerve are accomplished. The bony roof of the optic canal as well as its medial and lateral walls are carefully removed with a 3 mm diamond high-speed drill under constant irrigation to avoid thermal damage to the optic nerve. An incision on the optic sheath is made by observing the cerebrospinal fluid (CSF) drainage. The optic nerve becomes gently retractable with a number 7 Penfield dissector to some extent away from the carotid artery, to facilitate the aneurysmal neck exposure for clipping. Moreover, the optic nerve becomes decompressed as an angle away from the ICA is gained (Figures 4, 5).

The surgical technique of the optic foraminotomy has been described in detail in Supplementary Video 1.

## Results

The postoperative course was favorable in both cases without any added visual defect. In the CT angiography reconstruction in cases 1 and 2 (Figures 2C,D, 3C,D) we could check the complete obliteration of the aneurysms with adequate carotid permeability. In the postoperative neurological control, both patients improved their visual deficits.

## Discussion

### Anatomical Considerations

**Figure 6:**
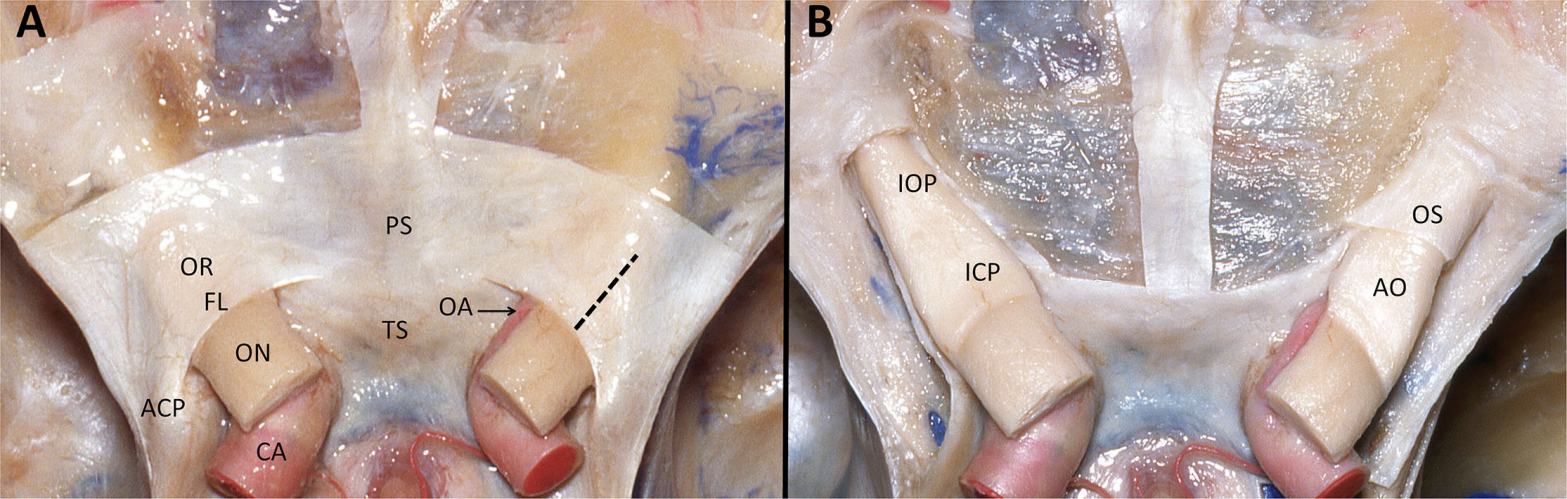
**(A)** Superior view of anterior and middle skull base dissection showing both internal carotid arteries in the supraclinoid segment (C6). We can observe the dura covering both optic nerve canals with its posterior concave border represented by the falciform ligament. The intracranial optic nerves were sectioned in order to show the trajectory of the carotid arteries. Laterally to the optic nerves and the carotid arteries are located the anterior clinoid processes. In dotted line is marked the distance to the free edge of the falciform ligament that we use to perform de durotomy. **(B)** After removing the dura and unroofing both optic canals, on the right optic nerve is exposed the first layer that corresponds to the optic sheath and internally the arachnoid of the optic nerve. OR, Optic roof; FL, Falciform ligament; ON, Optic nerve; CA, Carotid artery; ACP, anterior clinoid process; PS, Planum Sphenoidale; TS, Tuberculum sellae; OA, Ophthalmic artery; Dotted line, distance to the free edge of the falciform ligament that we use to perform de durotomy; IOP, Intraorbital optic nerve; ICP, intracanalicular optic nerve; OS, Optic sheath; AO, Arachnoid of the optic nerve.

Bouthillier et al. classified the internal carotid artery in seven segments: Cervical (C1), petrous (C2), lacerum (C3), cavernous (C4), clinoidal (C5), ophthalmic (C6), and communicant (C7) segments (16). The clinoidal segment was defined by the extradural segment in which the ICA stays between the proximal and distal dural rings, and below the anterior clinoid process (ACP). The ACP is a triangular osseous process that continues medially with the planum sphenoidale (10) and laterally with the lesser wing of the sphenoid bone. Its base corresponds to the optic strut that separates the optic canal from the superior orbital fissure and is generally pneumatized. It is completely covered by dura except for the inferior surface, where the carotid-oculomotor membrane is located. The supraclinoid portion of the ICA begins when the artery emerges from the distal dural ring at the roof of the cavernous sinus. It enters the intracranial cavity on the medial side of the ACP below the optic nerve and courses posterior, superior, and slightly lateral to reach the lateral side of the optic chiasm (Figure 6). The ICA bifurcates in the area below the anterior perforated substance at the medial end of the Sylvian fissure to give rise to the anterior and middle cerebral arteries. On its course, it gives rise to the ophthalmic, anterior choroidal, and posterior communicating arteries, as well as other small perforating branches including the superior hypophyseal arteries (21), which arise from the ophthalmic segment and extend to the infundibulum of the pituitary gland (Figure 1B). The latter usually arises from the medial one-third of the superior surface of the ophthalmic segment of the ICA immediately distal to the cavernous sinus, in the area below the optic nerve, and medial to the ACP (21). The intracranial segment of the ophthalmic artery is usually very short and commonly enters the optic foramen within 1 to 2 mm of its origin.

The optic nerves enter the optic canals medial to the ACP and above the ophthalmic arteries (21). The falciform ligament is a fold of dura that passes above the optic nerve proximal to the optic foramen. The optic nerve is surrounded by a dura corresponding to the optic sheath and bathed by cerebrospinal fluid. Under this dura, the optic nerve has an internal arachnoid layer (Figure 6). In general, the optic nerve within the canal is surrounded by a very thin bone.

### Technical Considerations

Carotid-ophthalmic aneurysms represent a true microsurgical challenge due to their anatomically close relationships with important surrounding structures such as the optic nerve and carotid artery.

Sometimes, visual deficits worsen after the clipping of aneurysms that arise from the ICA due to direct injury (26). It has been reported that postoperative vision deterioration might be caused by thermal injury to the optic nerve during drilling of the bony structures, contusion of the anterior optic pathway during dissection and traction, and/or compromised blood flow through the ophthalmic artery and retinal ischemia due to temporary occlusion (23, 27).

Endovascular intervention has been also described for these aneurysms. Because of the anatomical challenges with surgical approaches, paraclinoid aneurysms have been one of the most common indications for endovascular treatment. However, we support the direct surgical approach to achieve a definitive and complete aneurysm exclusion, as well as allowing optic nerve decompression, without the need for antiplatelet agents (1).

Extradural clinoidectomy was first pioneered by Dolenc et al. (4) for approaching intracavernous vascular lesions and was later modified and widely used for treating neurovascular diseases and brain tumors (28). Hauser et al. (29) treated an optic compression due to a carotid-ophthalmic aneurysm by unroofing of the optic foramen, while Drake et al. (7) performed a removal of the anterior clinoid through a subfrontal route. Iwabuchi et al. (6) described the unroofing of the optic canal in detail and reported 6 consecutive cases with good results. In his report, Iwabuchi et al. (6) described the opening and detaching of the dura leaving a dural flap reflected medially. In our opinion, this dural flap can be dangerous during the drilling of the roof of the optic canal because it can get rolled in the drill and produce optic nerve injuries. Since then, many papers have been reported supporting clinoidectomy and unroofing of the optic canal as the microsurgical approach for clipping carotid-ophthalmic aneurysms (1, 3, 4, 11, 28, 30).

In the past, before accurate knowledge and microsurgical techniques in skull base and vascular neurosurgery were achieved, carotid-ophthalmic artery aneurysms were classically treated with artery ligation and even associated with the need of sacrifice of the optic nerve for its operative resolution (9, 10, 31). Afterward, with the development and refinement of the microsurgical techniques and instruments, advanced approaches have taken place as the one we report in the present article; optic foraminotomy with the liberation of the optic nerve by sectioning the optic sheath with its posterior mobilization.

In the literature, the surgical technique for optic canal unroofing is widely described, however, we prefer to use the term “*optic foraminotomy*,” as we performed the drilling not only on the superior wall of the optic canal but also in the medial and lateral walls. The optic foraminotomy allows a safe visualization and incision of the optic sheath with the accomplishment of a gentle mobilization of the optic nerve away from the carotid artery. Besides allowing the correct neck visualization of the aneurysm for clipping, it also enables direct visualization of the ophthalmic artery. This maneuver allows a fast optic nerve decompression of the pressure produced by the aneurysm beating below this visual structure. This objective is adequately achieved by removing the bony and dural structures that fix the intracanalicular segment of the optic nerve.

We used the free edge of the falciform ligament as the landmark to perform a 10 mm incision of the dura since it is a fixed element. In contrast, the bony edge of the optic canal is not a constant limit because the optic roof is a variable morphological osseous structure.

Optic foraminotomy shows several advantages compared to the anterior clinoidectomy technique; first, the drilling is carried out over a flat surface, however, the surface of the anterior clinoid process is curved and there is a risk of injury to either the optic nerve or the ICA due to a technical failure. Next, optic foraminotomy enables a gentle nerve decompression as the optic nerve gains an angle away from the carotid artery and the aneurysm, as we have previously referred. By freeing the optic nerve, we can gently mobilize not only the optic nerve but also the ICA. With this technique, it is possible to dissect the arachnoid between the inferior surface of the optic nerve and the superior surface of the ophthalmic-carotid artery segment, and decompression of the superior wall of the artery is possible to do the clipping more comfortable. In contrast, after anterior clinoidectomy, the optic nerve remains compressed between the carotid-ophthalmic aneurysm and the bony structures of the optic canal. Moreover, optic foraminotomy can be performed much more quickly than anterior clinoidectomy, reducing surgical time and the surgeon's fatigue. In addition, the risk of bleeding is higher when performing clinoidectomy compared to optic foraminotomy, as this structure is adjacent to the cavernous sinus. Finally, optic foraminotomy is less traumatic because the optic nerve is covered by the optic sheath that protects it, as we showed in the anatomical specimens. Drilling of the medial part of the anterior clinoid process can produce a high risk of thermal injury of the clinoidal segment of the internal carotid artery; which is covered only by a thin layer of the carotid collar, or the ophthalmic artery; which arises from the internal carotid artery at the superomedial portion of the supraclinoid ICA and courses below the optic nerve. This anatomical relation provides safety during optic foraminotomy. Another risk that might be avoided by performing this microsurgical technique instead of the anterior clinoidectomy is the risk of damage of the oculomotor nerve.

In our experience, we have observed during surgical procedures, as well as in all the cadaveric specimens, an impression of the falciform ligament in the superior surface of the optic nerve even though there was no pressure on the optic nerve. We think that the explanation for this fact could be the carotid beat to the inferior surface of the optic nerve with the systolic and diastolic movements.

Optic foraminotomy is indicated in carotid-ophthalmic aneurysms <10 mm and those originating from the superior aspect of the ICA. Optic foraminotomy is also recommendable in other procedures, for instance when removal of meningiomas arising on the tuberculum sellae as one of the first surgical maneuvers. Conversely, anterior clinoidectomy is mandatory for large to giant superior paraclinoid aneurysms involving the origin of the ophthalmic artery, as well as for lateral projecting ones. Anterior clinoidectomy is also advantageous for ventral and medial paraclinoid aneurysms. In these aneurysms' types, anterior clinoidectomy allows the cut circumferentially the distal dural ring of the ICA and mobilization of the ICA itself, avoiding the potential sling effect after clipping. For larger ophthalmic aneurysms, an intradural clinoidectomy is recommended to achieve an early visualization of the dome in case of premature rupture. The main advantages of the anterior clinoidectomy lie in a more proximal control of the ICA, including the cavernous segment, and wider surgical freedom around the aneurysm neck. It must be stressed that a paramount aspect for the successful management of carotid-ophthalmic aneurysms, as well as other types of aneurysms, remains the constant neurovascular training (32–34).

The present report consists of a technical note and, accordingly, further studies have to be implemented to validate the clinical results of the proposed technique.

## Conclusion

In conclusion, we highly recommend optic foraminotomy since it is a safe, quick, and relatively easy technique for exposing and treating superior carotid-ophthalmic aneurysm and allowing decompression of the visual apparatus during the first stages of the procedure. It shows several advantages over the current anterior clinoidectomy technique regarding surgical exposure as well as facilitating visual improvement. With this technique, it is possible to dissect the arachnoid between the inferior surface of the optic nerve, and the superior surface of the ophthalmic-carotid artery segment and decompression of the superior wall of the artery is possible to do the clipping more comfortable.

## Data Availability Statement

The raw data supporting the conclusions of this article will be made available by the authors, without undue reservation.

## Ethics Statement

The studies involving human participants were reviewed and approved by IRB of San Fernando Hospital, Buenos Aires, Argentina. The patients/participants provided their written informed consent to participate in this study. This manuscript has been approved by our Hospital Ethical Committee and we have the explicit consent of the two patients for the publication of their photos and pre-postoperative studies.

## Author Contributions

All authors listed have made a substantial, direct, and intellectual contribution to the work and approved it for publication.

## Conflict of Interest

The authors declare that the research was conducted in the absence of any commercial or financial relationships that could be construed as a potential conflict of interest. The handling editor is currently organizing a Research Topic with the authors SL and AG.

## Publisher's Note

All claims expressed in this article are solely those of the authors and do not necessarily represent those of their affiliated organizations, or those of the publisher, the editors and the reviewers. Any product that may be evaluated in this article, or claim that may be made by its manufacturer, is not guaranteed or endorsed by the publisher.
